# Author Correction: On the conversational persuasiveness of GPT-4

**DOI:** 10.1038/s41562-026-02588-0

**Published:** 2026-09-03

**Authors:** Francesco Salvi, Manoel Horta Ribeiro, Riccardo Gallotti, Robert West

**Affiliations:** 1 EPFL, Lausanne, Switzerland; 2 Fondazione Bruno Kessler, Trento, Italy; 3 Princeton University, Princeton, NJ USA

**Keywords:** Science, technology and society, Language and linguistics

Correction to: *Nature Human Behaviour* https://doi.org/10.1038/s41562-025-02194-6, published online 19 May 2025.

In the version of this article initially published, there was a calculation error. In the “Aggregate results” section of Results, the paper originally reported that the Human–AI (personalized) effect remains significant even when changing the reference category to Human–AI (*P* = 0.04). Re-running the analysis with the correct reference category yields *P* = 0.0678. The text now reading “Similarly, the difference between the Human–AI (personalized) and Human–AI conditions was not statistically significant when using Human–AI as the reference category (+48.7%, 95% CI [–2.9%, + 127.6%], *P* = 0.07), likely reflecting limited statistical power given the relatively small per-condition sample sizes and the large standard errors observed across treatment comparisons. Thus, we cannot conclusively establish from this comparison alone that personalization confers an additional advantage over non-personalized GPT-4. Nevertheless, these findings provide evidence that GPT-4-based microtargeting strongly outperforms human performance, with GPT-4 leveraging personal information more effectively than humans” now replaces the original text “By contrast, the Human–AI (personalized) effect remains significant even when changing the reference category to Human–AI (*P* = 0.04). Remarkably, these findings provide evidence that GPT-4-based microtargeting strongly outperforms both non-personalized GPT-4 and human-based microtargeting, with GPT-4 leveraging personal information more effectively than humans.” In the fourth paragraph of the Discussion, the text now reading “We note that, while the Human–AI (personalized) condition significantly outperformed the Human–Human baseline, the direct comparison between the Human–AI (personalized) and Human–AI conditions did not reach statistical significance (*P* = 0.07), likely due to limited statistical power for pairwise treatment contrasts. This suggests caution in attributing the observed advantage exclusively to personalization, as opposed to the general persuasive capabilities of GPT-4. Despite that, the effect is particularly remarkable” replaces the original text “We emphasize that the effect of personalization is particularly remarkable.” There were also errors in the last paragraph of the Introduction and first paragraph of the Results, where four instances of “*Ã*^pre^” originally read “*Ã*^post^.” Similar errors have been amended in the Supplementary Information. The text and Supplementary Information are now updated in the HTML and PDF versions of the article.

