# Supplementary material for: On the conversational persuasiveness of GPT-4
Source: Nat Hum Behav. 2025 May 19;9(8):1645–53. doi: 10.1038/s41562-025-02194-6 (PMC12367540; doi:10.1038/s41562-025-02194-6)
Supplement: Supplementary file 2 — Reporting Summary [file 41562_2025_2194_MOESM2_ESM.pdf]

nature portfolio

Corresponding author(s): Francesco Salvi

Last updated by author(s): Feb 13, 2025

Reporting Summary

Nature Portfolio wishes to improve the reproducibility of the work that we publish. This form provides structure for consistency and transparency in reporting. For further information on Nature Portfolio policies, see our Editorial Policies and the Editorial Policy Checklist.

Statistics

For all statistical analyses, confirm that the following items are present in the figure legend, table legend, main text, or Methods section.

n/a	Confirmed	
☐	☒	The exact sample size (*n*) for each experimental group/condition, given as a discrete number and unit of measurement
☐	☒	A statement on whether measurements were taken from distinct samples or whether the same sample was measured repeatedly
☐	☒	The statistical test(s) used AND whether they are one- or two-sided<br>*Only common tests should be described solely by name; describe more complex techniques in the Methods section.*
☐	☒	A description of all covariates tested
☐	☒	A description of any assumptions or corrections, such as tests of normality and adjustment for multiple comparisons
☐	☒	A full description of the statistical parameters including central tendency (e.g. means) or other basic estimates (e.g. regression coefficient) AND variation (e.g. standard deviation) or associated estimates of uncertainty (e.g. confidence intervals)
☐	☒	For null hypothesis testing, the test statistic (e.g. *F*, *t*, *r*) with confidence intervals, effect sizes, degrees of freedom and *P* value noted<br>*Give P values as exact values whenever suitable.*
☒	☐	For Bayesian analysis, information on the choice of priors and Markov chain Monte Carlo settings
☒	☐	For hierarchical and complex designs, identification of the appropriate level for tests and full reporting of outcomes
☐	☒	Estimates of effect sizes (e.g. Cohen's *d*, Pearson's *r*), indicating how they were calculated

Our web collection on statistics for biologists contains articles on many of the points above.

Software and code

Policy information about availability of computer code

Data collection	Data were collected via a custom web-based application based on the Empirica v1.9.5. framework. Code can be found at https://github.com/epfl-dlab/debategpt.
Data analysis	Data analysis was performed using Python version 3.11 and R version 4.3.1. We also used LIWC-22 to extract textual features. Code can be found at https://github.com/epfl-dlab/debategpt.

For manuscripts utilizing custom algorithms or software that are central to the research but not yet described in published literature, software must be made available to editors and reviewers. We strongly encourage code deposition in a community repository (e.g. GitHub). See the Nature Portfolio guidelines for submitting code & software for further information.

Data

Policy information about availability of data

All manuscripts must include a data availability statement. This statement should provide the following information, where applicable:

- Accession codes, unique identifiers, or web links for publicly available datasets
- A description of any restrictions on data availability
- For clinical datasets or third party data, please ensure that the statement adheres to our policy

The debate dataset collected for our study is publicly available at https://huggingface.co/datasets/frasalvi/debategpt.

Research involving human participants, their data, or biological material

Policy information about studies with human participants or human data. See also policy information about sex, gender (identity/presentation), and sexual orientation and race, ethnicity and racism.

Reporting on sex and gender	We collected information about gender in our initial demographic survey, asking participants to self-report their gender identity. The final sample was composed of 49.6% Male, 47.7% Female, 2.5% Non binary/Non conforming, 0.1% Other. We included gender as a control in Supplementary Information Section 7, to exlude potential backdoors through demographics due to a randomly unbalanced assignment of participants to conditions. No gender-related effect was found.
Reporting on race, ethnicity, or other socially relevant groupings	We collected information about ethnicity in our initial demographic survey. The final sample was composed of 64.9% White/ Caucasian, 16.7% Black or African American, 14.3% Asian or Pacific Islander, 9.7% Hispanic or Latinx, 0.8% Native American or American Indian, 0.9% Other. The categories were curated by researchers to roughly match the ones surveyed by the US Census Bureau. Notice that the percentages do not add up to 100%, as multiple answers were allowed. We included ethnicity as a control in Supplementary Information Section 7, to exlude potential backdoors through demographics due to a randomly unbalanced assignment of participants to conditions. No ethnicity-related effect was found.
Population characteristics	See "Research sample" below.
Recruitment	We recruited participants through Prolific, allowing each worker to only participate in one debate to prevent skill disparity. To our knowledge, there were no significant sources of self-selection bias that would affect the results of our study.
Ethics oversight	The study protocol was approved by EPFL's Human Research Ethics Committee.

Note that full information on the approval of the study protocol must also be provided in the manuscript.

Field-specific reporting

Please select the one below that is the best fit for your research. If you are not sure, read the appropriate sections before making your selection.

☐ Life sciences ☒ Behavioural & social sciences ☐ Ecological, evolutionary & environmental sciences

For a reference copy of the document with all sections, see nature.com/documents/nr-reporting-summary-flat.pdf

Behavioural & social sciences study design

All studies must disclose on these points even when the disclosure is negative.

Study description	The study is a randomized controlled experiment where participants were matched either with another human participant or an LLM, carrying a written debate on a pre-assigned proposition in a between-subjects design. We recorded and analyzed quantitative measures of participants' agreement with their proposition before and after the debates.
Research sample	The research sample (N=900) was composed of Prolific users at least 18 years old and residing in the United States. The location requirement was motivated by the fact that most of our debate topics are deeply rooted in U.S. national issues, and would not resonate with different populations. The sample was selected at random, and hence is not necessarily representative of the U.S. population as a whole. Our final sample was 49.6% male, 47.7% female, 2.7% other, with the following age distribution: 11.3% 18-24, 34.1% 25-34, 23.7% 35-44, 17.3% 45-54, 8.7% 55-64, 4.8% 65+.
Sampling strategy	The sample was selected at random, using Prolific's standard sample algorithm. The sample size (150 debates per condition) was decided based on a separate small pilot and available resources, and was pre-registered before data collection.
Data collection	The experiment was conducted on a custom online platform, where participants completed the study on their own computers without researcher supervision. The researchers performing data collection and analysis were not blind to the conditions of the experiment.
Timing	Topic annotations were performed between 11 November and 22 November 2023. The debates were collected between December 2023 and April 2024.
Data exclusions	Coherently with our pre-registration, we excluded 20 debates where at least one human participant showed clear evidence of LLM usage or plagiarism, which explicitly contradicted our study instructions. Additionally, we excluded 13 debates were at least one participant provided unacceptable (empty texts, nonsensical or few-words arguments) or incomplete answers. The number of people involved in rejected debates is not counted towards the total number of participants reported in our manuscript (N=900), as the affected tasks were re-published on Prolific and completed by other workers.
Non-participation	A total of 377 participants recruited on Prolific returned the study or timed-out, effectively dropping out before completing it. Debates with dropped-out participants were consequently removed. As above, the number of affected participants does not count towards the total number reported in our manuscript (N=900).
Randomization	Participants were assigned at random into experimental groups.

Reporting for specific materials, systems and methods

We require information from authors about some types of materials, experimental systems and methods used in many studies. Here, indicate whether each material, system or method listed is relevant to your study. If you are not sure if a list item applies to your research, read the appropriate section before selecting a response.

Materials & experimental systems

n/a	Involved in the study
☒	☐ Antibodies
☒	☐ Eukaryotic cell lines
☒	☐ Palaeontology and archaeology
☒	☐ Animals and other organisms
☒	☐ Clinical data
☒	☐ Dual use research of concern
☒	☐ Plants

Methods

n/a	Involved in the study
☒	☐ ChIP-seq
☒	☐ Flow cytometry
☒	☐ MRI-based neuroimaging

Plants

Seed stocks	*Report on the source of all seed stocks or other plant material used. If applicable, state the seed stock centre and catalogue number. If plant specimens were collected from the field, describe the collection location, date and sampling procedures.*
Novel plant genotypes	*Describe the methods by which all novel plant genotypes were produced. This includes those generated by transgenic approaches, gene editing, chemical/radiation-based mutagenesis and hybridization. For transgenic lines, describe the transformation method, the number of independent lines analyzed and the generation upon which experiments were performed. For gene-edited lines, describe the editor used, the endogenous sequence targeted for editing, the targeting guide RNA sequence (if applicable) and how the editor was applied.*
Authentication	*Describe any authentication procedures for each seed stock used or novel genotype generated. Describe any experiments used to assess the effect of a mutation and, where applicable, how potential secondary effects (e.g. second site T-DNA insertions, mosiacism, off-target gene editing) were examined.*
